# Pericapsular nerve group block with phenol for non‐operative hip fracture management in patients living with frailty: a prospective multicentre cohort study

**DOI:** 10.1111/anae.70346

**Published:** 2026-09-08

**Authors:** Rachel J. H. Smits, Marte I. Lommerse, Hugo H. Wijnen, Lennart G. Wasmoeth, Glenn van de Vossenberg, Gezina T. M. L. Oei, Henk J. Schuijt, Kris C. P. Vissers, Hanna C. Willems, Jan Willem Kallewaard, Selina E. I. van der Wal, the Dutch‐PENG registry collaborators, Gert R. Roukema, Dawi Van der Stap, J.S.H.A. (Seppe) Koopman, Bart P.A. Spaetgens, Renée Brüggemann, Nicole Lefel, Manon Vugts, Nikki M.F. Noorda, Vincent Van Dongen, Hidde Krot, Nicole Grupa, Daisy Hoofwijk, Léon Timmerman

**Affiliations:** ^1^ Department of Anaesthesiology, Pain and Palliative Medicine Radboudumc Nijmegen the Netherlands; ^2^ Department of Trauma Surgery Amsterdam University Medical Centre Amsterdam the Netherlands; ^3^ Department of Internal Medicine, Geriatrics Section Amsterdam University Medical Centre Amsterdam the Netherlands; ^4^ Department of Geriatrics Rijnstate Hospital Arnhem the Netherlands; ^5^ Department of Anaesthesiology St. Antonius Hospitals Nieuwegein the Netherlands; ^6^ Department of Anaesthesiology Zuyderland Hospital Heerlen the Netherlands; ^7^ Department of Anaesthesiology and Pain Medicine Dijklander Hospital Hoorn the Netherlands; ^8^ Department of Anaesthesiology and Pain Medicine Rijnstate Hospital Arnhem the Netherlands; ^9^ Department of Anaesthesiology and Pain Medicine Amsterdam University Medical Centre Amsterdam the Netherlands

**Keywords:** acute pain management, frailty, hip fracture, neurolysis, pain intervention

## Abstract

**Introduction:**

The incidence of hip fractures is rising with the ageing population. For patients living with severe frailty and limited life expectancy, non‐operative management is increasingly considered after shared decision‐making. Given the limited availability of long‐term analgesic options, alternative techniques are needed. Chemical neurolysis via the pericapsular nerve group block approach can selectively target sensory innervation of the hip.

**Methods:**

A prospective multicentre cohort study was conducted at seven Dutch hospitals. Patients who chose non‐operative management for a hip fracture and met the indication for neurolysis were enrolled. Patient‐reported outcomes were assessed at 24 h, 1 week, 30 days and 3 months and included: quality of life; pain; analgesic use; mobility; complications; and mortality.

**Results:**

We included 103 patients (mean (SD) age 87 (6.4) y); 51% were nursing home residents and 76% had cognitive impairment. The pericapsular nerve group block was performed under ultrasound guidance, with second needle placement in 14/103 (14%) patients. Mean (SD) injected volume of phenol 6% was 10 (3.1) ml. At 1 week, 34/36 (94%) patients reported acceptable pain at rest and 47/53 (89%) reported no or acceptable pain when washing and dressing. A bed‐chair transfer was possible in 24/53 (45%) patients at 1 week. No procedure‐related adverse events were reported. Mortality rate was 72/91 (79%) after 3 months, with a 30‐day mortality of 56/91 (62%) and median (IQR [range]) survival of 9 (6–24 [0–85]) days.

**Discussion:**

The pericapsular nerve group block with phenol 6% is an effective, minimally invasive procedure for pain management in addition to opioid‐based pain relief in patients living with frailty, when surgery is not chosen. Procedure standardisation may improve clinical outcomes.

## Plain Language Summary

**What we did:**

We observed a group of older people with a broken hip that did not have surgery. These people got a procedure to block the nerves going to the hip joint (called the ‘PENG block’). We studied the effect of this block on the quality of life, their pain at rest and with movement, the need for pain medicines and how satisfied they were with this block. We also observed how well they could move in the weeks after the block, if they got problems like infection, and how long they lived.

**Why we did it:**

Older adults can have trouble walking and other health problems, like diseases of the heart, lungs, or brains. Sometimes these problems make surgery too risky, and a decision can be made to not do surgery. Without surgery, a broken hip can hurt a lot, especially when moving. By blocking the nerves with the ‘PENG block’, we try to make the pain easier to bear so people can be more comfortable.

**What we found:**

We studied 103 people with a broken hip, with an average age of 87 years. We found good pain relief with the combination of the ‘PENG block’ and pain medicines in most people. This was both at rest as during daily care, such as washing and dressing. About half could move to a chair at 1 week, and about two‐thirds after 4 weeks. Most people were fairly satisfied with the treatment. Many people had a short life after a broken hip without surgery; 61% died within 30 days and 79% died within 3 months. This means that the PENG block can help reduce pain and help some people move a bit more, but people who do not have surgery after a broken hip often have a short time left.

## Introduction

Hip fractures represent a significant global health concern and the incidence is rising with the ageing population [1, 2]. Patients with multiple comorbidities, cognitive impairment and limited mobility are at increased risk of peri‐operative complications and mortality [3]. For patients living with severe frailty, limited life‐expectancy and for whom surgery is not aligned with their goals of care, non‐operative management may be an alternative, offering comparable quality‐of‐life outcomes [4, 5].

Since 2016, the Netherlands has a national hip fracture registry to monitor and improve quality of care, in which 66/68 hospitals are participating. Co‐management by surgeons and geriatricians is a key indicator and was achieved in almost 80% of hip fracture cases by 2020 [6]. This has led to more consistent multidisciplinary shared decision‐making processes, despite persistent variability in care pathways [7]. Implementation studies and protocols have supported these collaborative processes and emphasised attention in this vulnerable population [8]. Advance care planning is practised widely in the Netherlands, typically led by general practitioners, geriatricians or physicians working with nursing home residents. This conversation often extends beyond resuscitation status and includes broader goals of care, for example when the patient sustains a hip fracture [9, 10]. Up to 8% of patients sustaining a hip fracture in the Netherlands choose non‐operative management [11].

When opting for non‐operative management, the focus shifts towards end‐of‐life care and adequate pain management [4]. Opioids are often needed but are associated with considerable adverse effects [12]. Loco‐regional nerve blocks with a catheter can be an alternative in pain management but are not suitable for long‐term pain relief outside a hospital inpatient setting [13, 14]. Due to the lack of effective, long‐term options, different techniques are needed for additional pain relief in patients living with frailty.

The pericapsular nerve group (PENG) block was developed based on a cadaveric anatomical study examining the innervation of the anterior hip capsule [15]. The anterior hip capsule is innervated by sensory branches of the femoral nerve, obturator nerve and, often, the accessory obturator nerve, with predominantly nociceptive input [16]. The PENG block involves visualisation of the anterior inferior iliac spine and the iliopubic eminence, targeting the fascial plane beneath the iliopsoas muscle [17]. Given the sensory nature of the innervation of the anterior hip capsule, it was considered a potential target for chemical neurolysis [18, 19]. In a previous retrospective cohort study, patients receiving a PENG block with phenol achieved acceptable pain scores alongside reduced opioid consumption [20]. Patients and families report overall satisfaction despite issues with periprocedural logistics, communication and adjunct analgesia [21]. Follow‐up was limited because a considerable number of patients are discharged back to their referring nursing homes in the Netherlands after neurolysis [21].

To address these gaps and to prospectively evaluate the outcome of the clinical practice in the Netherlands, we conducted a multicentre cohort study. We hypothesised that ultrasound‐guided PENG block with phenol would provide effective and sustained analgesia, with improved quality of life, in patients living with frailty who were managed non‐operatively following hip fracture. We aimed to provide a standardised clinical pathway that optimises patient selection, informed consent and adjunct pain management.

## Methods

This prospective cohort study was conducted in five general and two academic teaching hospitals in the Netherlands. The requirement for medical ethical approval was waived by the medical ethics committee and the study was registered at each participating hospital, according to local regulations.

Patients with non‐operative management for a femoral neck or trochanteric fracture were enrolled. The decision to pursue non‐operative management followed a multidisciplinary shared decision‐making process with patients and/or proxies and considering factors such as frailty; peri‐operative risk assessment; and life‐expectancy. After the indication for additional pain relief via a PENG block with phenol was confirmed, patients were included and written informed consent by the patient or a proxy was obtained. Patients with subtrochanteric, bilateral and periprosthetic fractures were not studied.

The PENG block was performed via an ultrasound‐guided injection [17]. The anterior iliac spine, iliopubic eminence and psoas tendon were visualised. A needle was placed laterally from the psoas tendon. After verification of the correct needle placement, phenol 6% in an aqueous solution was injected. At retraction, the needle was flushed with either 0.9% saline or a local anaesthetic to prevent damage of the surrounding tissue due to leakage of phenol 6% in the adjacent subcutaneous tissue. In some cases, a second injection was performed to selectively target the branches of the obturator nerve at the inferomedial acetabulum.

The following details were noted: technique used (one needle or two needle placements); amount of aqueous phenol 6%; additives (0.9% saline, local anaesthetics); difficulty level (easy, moderate or difficult); level of pain directly after the intervention; and complications due to the procedure.

Pre‐interventional assessment consisted of demographic data from the electronic patient files, activities of daily living according to the Katz ADL index [22] and the EuroQol 5‐Dimension 5‐Level Questionnaire (EQ5D‐5L) [23].

Patient characteristics collected included: age; sex; BMI; type of fracture; ASA physical status score; Charlson comorbidity index [24]; pre‐existing opioid use and daily dosage in oral morphine equivalents (OME); use of anticoagulants; Short Nutritional Assessment Questionnaire; presence of cognitive impairment or diagnosis of dementia; living situation (home or in a nursing home); and Functional Ambulation Category score (a six‐point scale to classify walking ability).

The primary outcome was the health‐related quality of life, measured using the EQ5D‐5L. The secondary outcome was the level of pain reported on a numeric rating scale (NRS) or the Pain Assessment Checklist for Seniors with Limited Ability to Communicate – Dutch (PACSLAC‐D) [25], depending on the cognitive status of the patient. Because of the non‐interventional nature of the study, patients were not mobilised actively for pain assessment; scores were observed during daily activities, care or repositioning of the patient. To assess pain during movements required for daily care, patients or their proxies were asked whether pain was acceptable during washing and dressing. Other secondary outcomes included: opioid consumption in OME; treatment satisfaction on a scale from 0 (extremely dissatisfied) to 10 (extremely satisfied); mobility on a 4‐point scale (bedbound, bed‐chair transfer, few steps (action radius < 10 m), mobile (action radius > 10 m)); mortality; time to death; and the quality of dying via the Quality of Dying and Death questionnaire [26]. Complications included: delirium (clinical diagnosis); pressure ulcer (clinical diagnosis); anaemia (haemoglobin decrease > 16 g.l^‐1^); heart failure (new clinical diagnosis or increased dosage of furosemide); decline of renal function (eGFR decrease > 20%); pneumonia (infiltration on radiograph); and urinary tract infection (positive urine culture). Complications were only registered when clinically diagnosed; no additional diagnostic procedures were performed. Level of pain and treatment satisfaction were scored on day 1. Assessment of all primary and secondary outcomes was performed at 1 week, 30 days and 3 months.

All data were registered in an electronic database using CASTOR EDC (Amsterdam, the Netherlands). All analyses were conducted using SPSS (v. 29, IBM, Armonk, NY, USA). A univariate regression analysis was performed to describe the relation between clinical variables and the EQ5D‐5L pain component at 1 week, and the treatment satisfaction (0–10) at 24 h and 1 week. The effect (β) is presented with a 95%CI. p values < 0.05 were considered statistically relevant.

## Results

Between 18 August 2023 and 28 February 2025, we identified 106 patients managed non‐operatively for a hip fracture; three were not studied because of missing demographic data (Fig. 1). Non‐operative management was chosen after multidisciplinary assessment in 93/103 (92%). Consent was obtained through a proxy in 83/103 (81%) patients. The characteristics of the 103 included patients reflected their frailty status with a mean (SD) age of 87 (6.4) y, mean (SD) Charlson Comorbidity Index of 4.2 (2.6) and cognitive impairment present in 78/103 (76%). In total, 76/103 (74%) sustained a femoral neck fracture and 27/103 (26%) a trochanteric fracture (Table 1).

**Figure 1:**
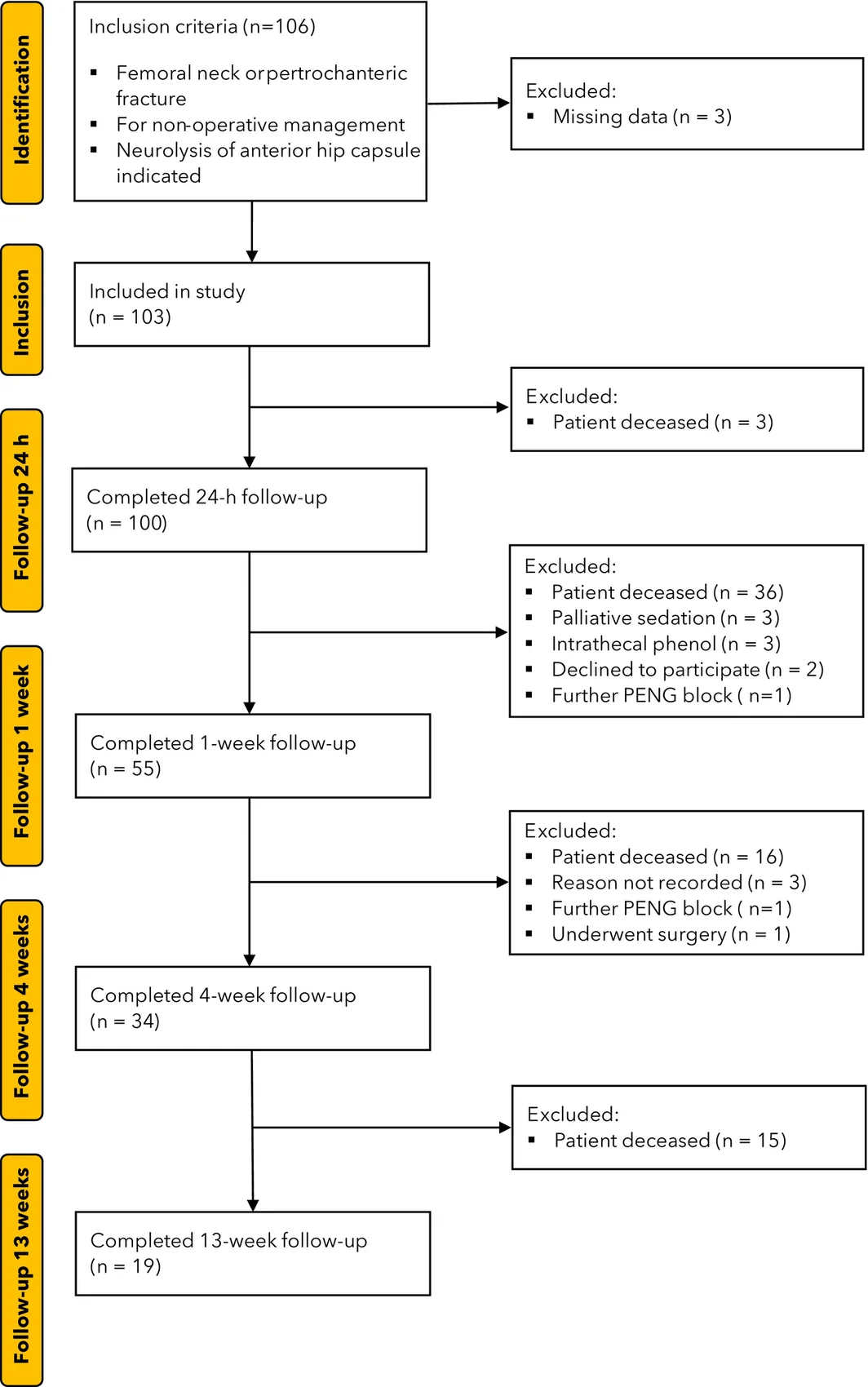
Study flow chart.

**Table 1:** Characteristics of the patient population. Values are mean (SD), number (proportion), mean (SD) or median (IQR [range]).

	n = 103
Age; y	87 (6.4)
Sex; male	40 (39%)
BMI; kg.m^‐2^	23.0 (4.0)
ASA physical status	
2	4 (4%)
3	56 (54%)
4	42 (41%)
5	1 (1.0%)
Charlson comorbidity index	4.2 (2.6)
Pre‐admission opioid use	14 (14%)
Anticoagulant/antiplatelet use	
Direct oral anticoagulants	16 (16%)
Vitamin K antagonist	11 (11%)
Platelet aggregation inhibitor	28 (28%)
Low molecular weight heparin	3 (3%)
None	43 (43%)
Missing	2
**Functional status and nutrition**	
Activities of daily living* score	
A	10 (10%)
B	3 (3%)
C	20 (19%)
D	21 (20%)
E	10 (10%)
F	20 (19%)
G	11 (11%)
H	7 (7%)
Missing	1
Nutritional assessment (SNAQ)	
Low risk of malnutrition (≤ 1)	78 (76%)
Moderate malnutrition (2)	10 (10%)
Severe malnutrition (≥ 3)	15 (15%)
Dementia	63 (61%)
Cognitive disorders	
Yes	78 (76%)
No	22 (21%)
Unknown	3 (3%)
Nursing home residence:	53 (51%)
Immobile or Functional Ambulation Category score < 2**	28 (27%)

SNAQ, Short Nutritional Assessment Questionnaire.

* According to the Katz Index of Independence in Activities of Daily Living.

** A score < 2 indicates non‐functional ambulator or dependent on continuous physical assistance.

Descriptive data on the intervention are shown in Table 2. Two patients reported positioning‐related pain. All interventions were performed under ultrasound guidance. The classic PENG approach with one needle position was used in 89/103 (86%) interventions and a second needle was placed in 14/103 (14%). The mean (SD) injected volume of phenol 6% was 10 (3.1) ml. In 84/103 (82%) patients, additional liquid was used to perform hydrodissection, with a median (IQR [range]) volume of 2 (0.5–4 [0–20]) ml. No other immediate postprocedural complications occurred. After 15 min, 37/103 (36%) patients were able to rate their pain, with a median (IQR [range]) NRS pain score at rest of 2 (0–3 [0–8]); 32/37 (86%) reported a NRS score ≤ 4.

**Table 2:** Characteristics of the performed intervention. Values are median (IQR [range]), number (proportion) or mean (SD).

	n = 103
Time to intervention; h	30 (22–48 [5–249])
Difficulty level	
Easy	72 (70%)
Moderate	29 (28%)
Difficult	2 (2%)
Technique	
1 needle	89 (86%)
2 needles	14 (14%)
Quantity of phenol; ml	10 (3.12)
Other liquid used	
None	19 (18%)
0.9% saline	29 (28%)
Lidocaine	7 (7%)
Ropivacaine	45 (44%)
Bupivacaine	3 (3%)
Quantity of other liquid used; ml	2 (1–4 [0–20])
Quantity of other liquid used; > 5 ml	11 (11%)
Complications	
None	101 (98%)
Pain related to intervention	2 (2%)
Pain score 15 min after intervention	
NRS possible	37 (36%)
NRS	2 (0–3 [0–8])
NRS ≤ 4	32 (86%)

NRS, numerical rating scale.

Pre‐ and postprocedural pain scores are summarised in Figure 2 and Table 3. All outcomes are single measurements at the assessed time‐point. At 24 h, 100 patients were alive and data were available for 98. Thirty‐two patients completed the NRS, with a median (IQR [range]) of 2 (0–3.75 [0–8]) at rest and 6 (4–7 [1–9]) during movement. After 24 h, the PACSLAC‐D was assessed in 66 patients. Pain at rest was acceptable in 51/66 (77%) patients and unacceptable in 12/66 (18%) patients with a score during movement of 6 (3–8 [0–16]). Treatment satisfaction at 24 h was 6 (4–8 [0–10]). Median (IQR [range]) OME was 15 (8–27 [0–196]) mg per 24 h. Within 1 week, 70 patients were discharged, with an overall duration of hospital stay of 3 (2–6 [0–21]) days.

**Figure 2:**
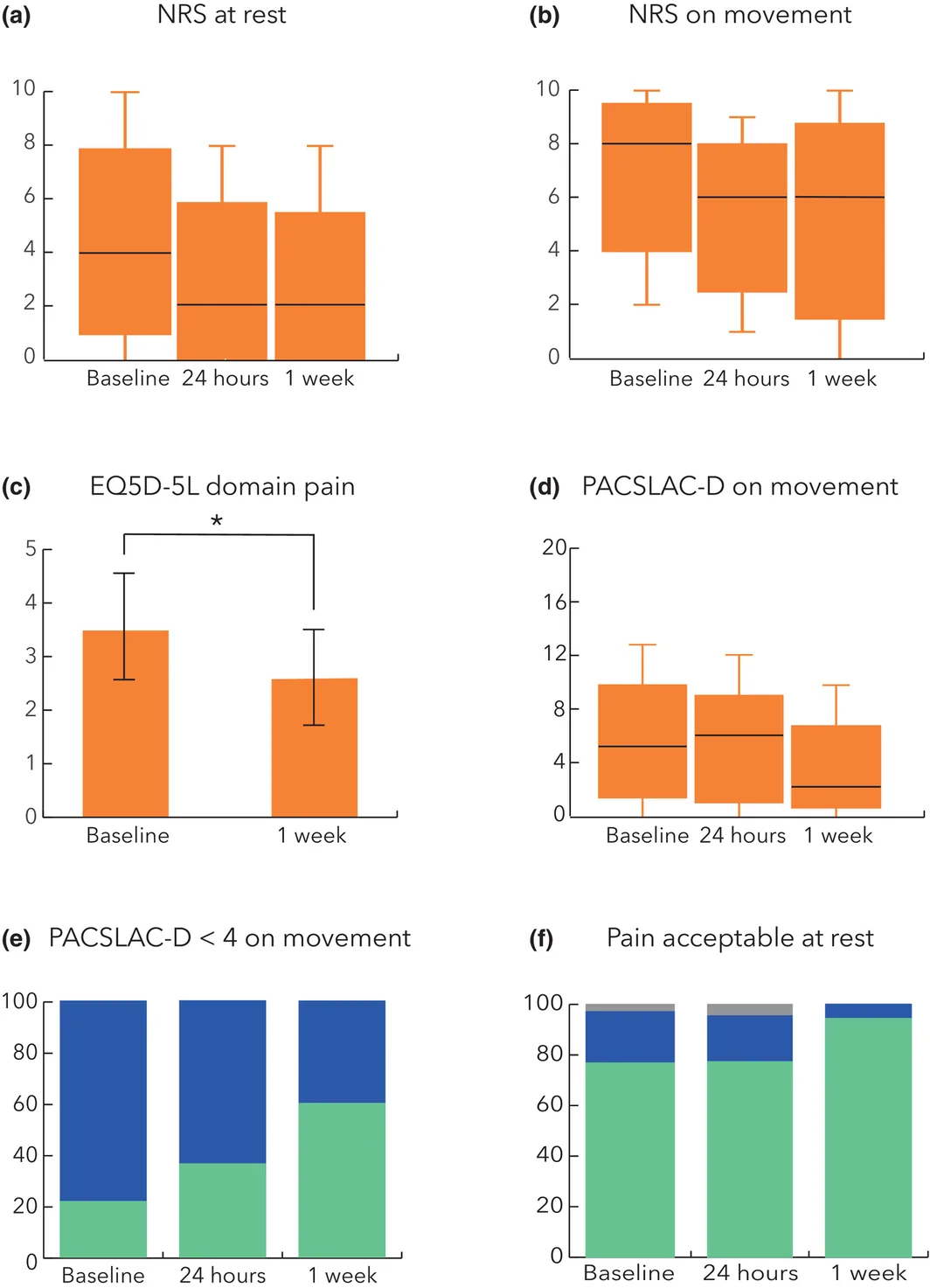
Results for pain at rest and with movement pre‐intervention, after 24 h and 1 week, assessed with numeric rating scale (a, b); EQ5D‐5L (c); PACSLAC‐D (d, e); percentage (f). (Data are median with error bars showing interquartile range, mean with error bars showing standard deviation or proportion (%), with yes (green), no (blue), or unknown (grey). EQ5D pain domain (*) shows a significant difference between groups (p < 0.001). NRS, Numeric rating scale; EQ5D‐5L, EuroQol 5‐Dimension 5‐level questionnaire; PACSLAC‐D, Pain Assessment Checklist for Seniors with Limited Ability to Communicate – Dutch.

**Table 3:** Postinterventional outcomes at different measurements. Values are mean (SD), median (IQR [range]) or number (proportion).

Data available/alive	24 h	1 week	30 days	3 months
	n = 98/100	n = 53/55	n = 30/34	n = 19
Numerical rating scale	n = 32	n = 17	n = 12	n = 9 (47%)
At rest	2 (0–4 [0–8])	2 (0–3 [0–8])	2 (0–5 [0–8])	2 (0–6 [0–6])
With movement	6 (4–7 [1–9])	6 (3–8 [0–10])	6 (4–8 [1–9])	4 (2–8 [0–9])
PACSLAC‐D	n = 66	n = 36	n = 18	n = 10
With movement	6 (3–8 [0–16])	2 (2–5 [0–13])	2.5 (1–6 [0–11])	1 (0–3 [0–4])
< 4	24 (36%)	21 (58%)	10 (56%)	8 (80%)
Pain acceptable at rest				
Yes	51 (77%)	34 (94%)	18 (100%)	8 (80%)
No	12 (18%)	2 (6%)	0	1 (10%)
Unknown	3 (5%)	0		1 (10%)
Pain during washing and clothing				
No pain		4 (8%)	5 (17%)	9 (47%)
Pain, but acceptable		43 (81%)	20 (67%)	8 (42%)
Pain, not acceptable		6 (11%)	5 (17%)	2 (11%)
Treatment satisfaction; 0–10	6 (4–8 [0–10])	6 (5–8 [2–10])	7 (5–8 [2–10])	8 (6–9 [1–10])
Treatment satisfaction ≥ 6	54 (63%)	36 (68%)	21 (70%)	15 (79%)
Analgesia				
None		4 (9%)	2 (8%)	2 (13%)
Paracetamol		6 (13%)	5 (21%)	4 (25)
Opioid		37 (79%)	17 (71%)	10 (62.5%)
Missing		8	10	3
Oral morphine equivalents; mg day^‐1^	15 (8–27 [0–196])	30 (13–58 [0–225])	30 (11–69 [0–144])	15 (4–52 [0–165])
Missing	1	8	6	2
Mobility				
Bedbound		28 (53%)	11 (37%)	2 (10%)
Bed–chair transfer		24 (45%)	18 (60%)	14 (74%)
Few step		1 (2%)	1 (3%)	3 (16%)
Complications				
None	73 (73%)	35 (66%)	16 (53%)	12 (63%)
Delirium	16 (16%)	5 (9%)	4 (13%)	1 (5%)
Pressure ulcer	5 (5%)	11 (21%)	10 (33%)	6 (32%)
Pneumonia	1 (1%)	2 (4%)	2 (7%)	
Urinary tract infection	4 (4%)	2 (4%)		1 (5%)
Heart failure	2 (2%)			
Loss of kidney function	1 (1%)			

PACSLAC‐D, Pain Assessment Checklist for Seniors with Limited Ability to Communicate – Dutch.

At 1 week, 55 patients were alive and data were available for 53. Mean (SD) EQ5D‐5L pain score was 2.6 (0.95). A NRS for pain was obtained in 17/53 (32%) patients, with a median (IQR [range]) of 2 (0–3 [0–8]) at rest and 6 (3–8 [0–10]) with movement. The PACSLAC‐D was scored in 36/53 (68%) patients, with acceptable pain at rest reported in 34/36 (94%) and a score of 2 (2–5 [0–13]) with movement. During washing and dressing, 4/53 (8%) patients reported no pain, 43/53 (81%) rated the pain as acceptable and 6/53 (11%) as unacceptable. Treatment satisfaction was 6 (5–8 [2–10]). Opioid use was present in 37/47 (79%) patients, with an OME of 30 (13–58 [0–225]) mg in the previous 24 h. At 1 week, 28/53 (53%) patients were bedbound, 24/53 (45%) were able to manage a bed–chair transfer and one patient walked a few steps. In 35/53 (66%) patients, no complications were reported.

At 30 days, 34 patients were alive and data were available for 30. The mean (SD) EQ5D‐5L pain score was 2.5 (0.97). A NRS for pain was obtained in 12/30 (40%) patients, with a median (IQR [range]) of 2 (0–5 [0–8]) at rest and 6 (4–8 [1–9]) with movement. The PACSLAC‐D was scored in 18/30 (60%) patients, with acceptable pain at rest reported in 18/18 (100%) and a score of 3 (1–6 [0–11]) with movement. During washing and dressing, 5/30 (17%) patients reported no pain, 20/30 (67%) rated the pain as acceptable and 5/30 (17%) as unacceptable. Treatment satisfaction was 7 (5–8 [2–10]). Opioid use was present in 17/24 (71%) patients, with an OME of 30 (11–69 [0–144]) mg in the previous 24 h. At 30 days, 11/30 (37%) patients were bedbound, 18/30 (60%) were able to manage a bed–chair transfer and one patient walked a few steps. In 16/30 (53%) patients, no complications were reported.

At 3 months, 19 patients were alive and participating in the cohort. The mean (SD) EQ5D‐5L pain score was 2.1 (1.3). A NRS for pain was obtained in 9/19 (47%) with a median (IQR [range]) of 2 (0–6 [0–6]) at rest and 4 (2–8 [0–9]) during movement. The PACSLAC‐D was scored in 10/19 (53%) patients, with acceptable pain at rest reported in 8 (80%) and a score of 1 (0–3 [0–4]) with movement. During washing and dressing, 9/19 (47%) patients reported no pain, 8/19 (42%) rated the pain as acceptable and 2/19 (11%) as unacceptable. Treatment satisfaction was 8 (6–9 [1–10]). Opioid use was present in 10/16 (62.5%), with an OME of 15 (3.75–51.5 [0–165]) mg in the previous 24 h. At 3 months, 2/19 (10%) patients were bedbound, 14/19 (74%) were able to manage a bed–chair transfer and 3 (16%) patients walked a few steps. In 12/19 (63%) patients, no complications were reported at 3 months.

Detailed descriptives of the outcome and complications are shown in Table 3. Mobility and the self‐care ability remained low according to the EQ5D‐5L throughout the follow up. Full results of the EQ5D‐5L are shown in Figure 3.

**Figure 3:**
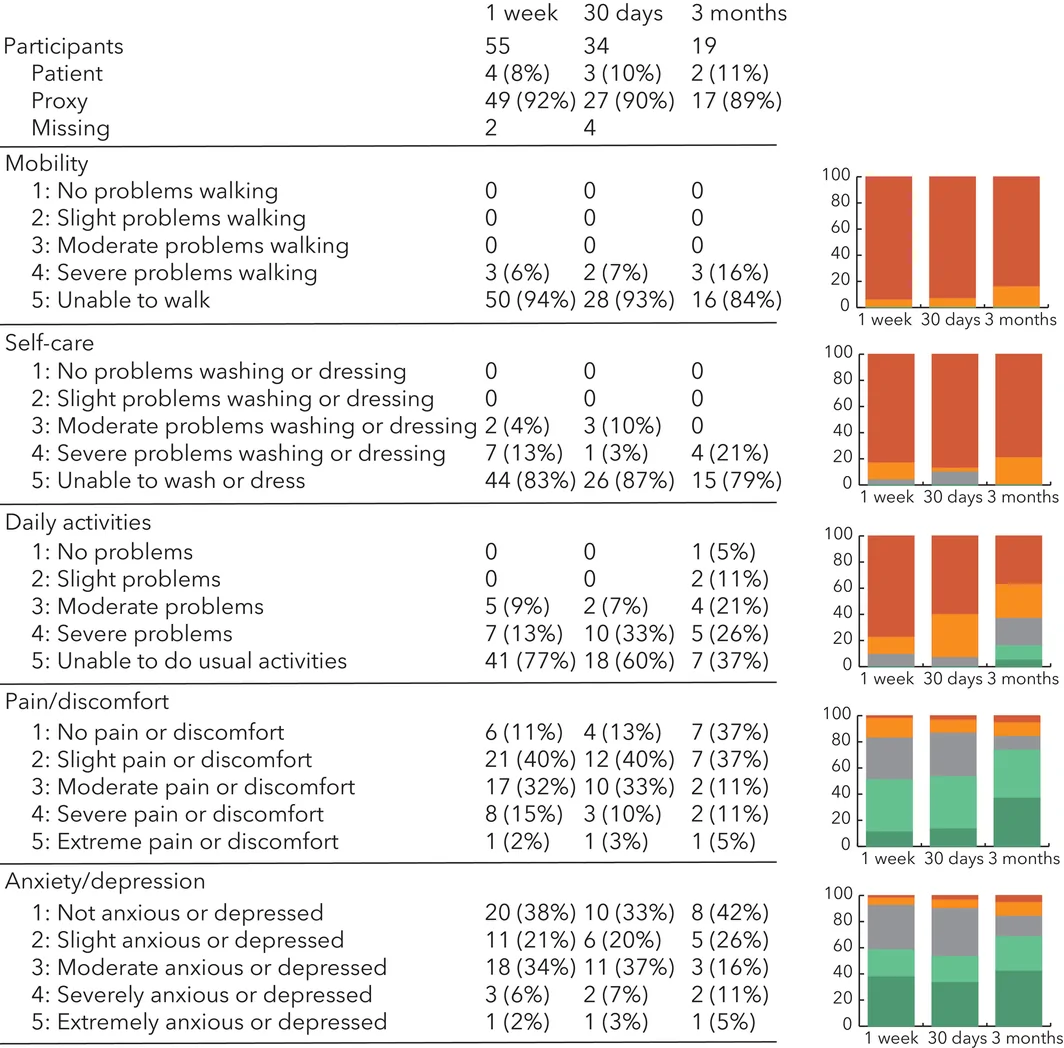
Outcome on the different EQ5D‐5L domains in numbers (proportion), with a score of 1 (dark orange); 2 (orange); 3 (grey); 4 (green); and 5 (dark green).

Mortality data were available in 91 patients; 72 (79%) died within 3 months, with a median (IQR [range]) survival of 9 (6–24 [0–85]) days. Mortality at 1 week was 28/91 (31%) and 30‐day mortality was 56/91 (62%) (online Supporting Information Tables S1 and S2). There was no difference in mortality rate between men and women (online Supporting Information Figure S1). Three patients underwent an alternative invasive procedure with intrathecal administration of phenol and two patients received a repeat PENG block with phenol. One patient improved clinically and proceeded to surgery after 1 week (Fig. 1).

Univariate regression analysis (online Supporting Information Table S3) showed that treatment satisfaction was lower at 24 h at one centre (β ‐3.26, 95%CI ‐5.85 to ‐0.66). Higher pre‐fracture opioid use correlated negatively with satisfaction. Patients using platelet aggregation inhibitors reported greater satisfaction than those using direct oral anticoagulants (β 1.92, 95%CI 0.23–3.61). Satisfaction was also higher in patients with femoral neck fractures and in those where the intervention was rated ‘moderate’ rather than ‘easy’. Each millilitre of phenol 6% was associated with higher satisfaction by 0.19 (95%CI ‐0.02–0.40) points. In contrast, ropivacaine use and larger volumes of additional liquids reduced satisfaction at 24 h.

There was a significant association between lidocaine use and EQ5D‐5L pain and treatment satisfaction at 1 week. Compared with no additional liquid, lidocaine was associated with increases in EQ5D‐5L pain scores by 1.40 (95%CI 0.47–2.34) and reduced satisfaction by 3.13 (95%CI ‐5.30 to ‐0.95) points. No other patient or intervention characteristics were significantly associated with EQ5D‐5L or satisfaction at 1 week. No associations were found between satisfaction and sex; Charlson comorbidity index; dementia; or mobility.

## Discussion

This multicentre prospective cohort study shows that anterior hip capsule neurolysis via the PENG block can serve as an effective minimally invasive procedure for pain management in addition to opioid‐based pain relief in patients living with frailty, when non‐operative management is preferred. Neurolysis with PENG block reflects current practice in the participating centres in the Netherlands, as the first step in the non‐operative management for selected patients. The decision for non‐operative management was weighed carefully in a shared decision‐making process with patients and/or proxies, based on multidisciplinary evaluation, encompassing frailty and peri‐operative risk assessment. The high mortality rate in this study reflects the frailty of this patient group and the choice for non‐operative management.

After 1 week, 89% of patients reported absent or acceptable pain during washing and dressing. Other large cohorts of patients having hip capsule neurolysis reported satisfactory pain relief in 65–71% [20, 27]. In our study, the PACSLAC‐D scores were higher at 24 h than at 1 week, possibly reflecting discomfort or trauma‐related haematomas. Opioid consumption was common; 79% received opioids after 1 week, declining thereafter. Daily OME was 15 mg on day 1 and 30 mg at 1 week and at 30 days; this is higher than in a retrospective cohort study of chemical denervation (7.5 mg at day 1, 15 mg at day 3) [20]. The use of opioids could be explained by progressive insight that neurolysis alone often might not be sufficient. After a fall, the hip fracture may not be the only source of pain and multimodal analgesia is commonly necessary to achieve adequate relief, particularly during mobilisation and routine care. Therefore, the study protocol permitted supplemental opioid prescription when the PENG block provided suboptimal analgesia. Although opioid consumption at 1 week was substantial (daily OME 30 mg), the dosage is significantly lower compared with a previous study on non‐operative management without the PENG block (daily OME 68 mg) [28].

We observed no significant difference in pain relief or treatment satisfaction at 1 week between femoral neck and trochanteric fractures. These findings indicate that the PENG block may also be effective for trochanteric fractures, consistent with cohorts on neurolysis of the anterior hip capsule [20, 27]. However, around 40% of hip fractures in the Netherlands are trochanteric, whereas only 26% were trochanteric in our study [29]. This suggests that pre‐enrolment selection for chemical neurolysis based on fracture type may have introduced selection bias.

Mobility was low, consistent with previous reports in which 50% of patients were able to sit after 5 days and 15% were able to walk with assistance after 4 months [18]. In our cohort, 47% achieved bed‐to‐chair transfer or a few steps after 1 week. No patients were able to mobilise > 10 m at any time point.

Previous studies reported a 70% complication rate; pneumonia (16%) and urinary tract infections (14%) were most common [28]. Complication rates were lower in our cohort, with delirium and pressure ulcers being observed most frequently. This may represent an underestimation, as complications were only recorded when diagnosed clinically. Apart from transient discomfort in two patients, no intervention‐related adverse events occurred.

European guidelines classify the PENG block as a deep fascial plane block and recommend discontinuation of anticoagulant therapy, though this recommendation remains contested [30, 31, 32]. The Dutch guideline does not address the PENG block specifically, but comparable ultrasound‐guided interventions are regarded as low risk and do not require the cessation of anticoagulants [33]. Despite anticoagulant use in 57% of patients (27% on direct oral anticoagulants or vitamin K antagonists), no haemorrhagic events were observed; this is consistent with other reports [20, 27]. At 24 h post‐procedure, patients on antiplatelet therapy reported greater satisfaction than those using direct oral anticoagulants. A possible explanation is that direct oral anticoagulant use may be associated with larger post‐procedural haematoma formation, producing more discomfort or myalgia. This hypothesis requires further prospective evaluation.

The PENG block may cause possible weakness due to aberrant spread of phenol to the femoral nerve, particularly when higher volumes were used [17]. Although quadriceps weakness was not actively assessed in our study, cadaveric data suggest femoral nerve involvement may occur at volumes > 13.2 ml [34].

The 30‐day mortality rate of 62% and 3‐month mortality rate of 79% likely reflects the advanced age of the cohort; high ASA physical status and Charlson comorbidity index scores; and high incidence of cognitive impairment (76%) and pre‐admission nursing home residence (51%). All of these are recognised risk factors for mortality after hip fracture [3, 28, 35]. No sex differences were observed in our cohort, despite male sex being reported as a risk factor [3, 28, 35]. A recent systematic review found an estimated 30‐day mortality ranging from 18% to 34% in non‐operative management, with a greater risk of death compared with operative management. Our cohort was, by definition, a group at high risk of early mortality.

There is a profound variation globally in the incidence and patient characteristics in non‐operative management for hip fracture, which is influenced by economical and geographical resources [36]. A recent analysis in Australia and New Zealand reported 30‐day mortality of 87% after palliative non‐operative care for patients with a hip fracture [37]. Comparable Dutch cohorts found a 30‐day mortality of 83% and 87% in patients managed non‐operatively, and 25% in those who had surgery [4, 38]. Median survival was 9 days in our cohort, comparable with the retrospective cohort of Kroes et al. [20], but slightly longer than in the observed 7 days in the FRAIL‐HIP study [4]. Differences in mortality may reflect improved mobilisation from better pain relief, or demographic variation, as non‐operative management has been extended to a broader population in the Netherlands in recent years [11, 29]. Interest in non‐operative hip fracture care in the Netherlands increased after the publication of the FRAIL‐HIP study [4]. A national multidisciplinary consortium was established to share experiences and clinical questions. This led to adjustments in previous care pathways and increased clinical and scientific collaborations, aiming to improve non‐operative hip fracture care and promoting its implementation across the country.

The FRAIL‐HIP study reported outcomes in a comparable cohort of patients managed operatively vs. non‐operatively for hip fracture, before the introduction of neurolysis via the PENG block [4]. At 1 week, severe or extreme pain on the EQ5D‐5L was present in 28–30% of patients managed non‐operatively, and in 7–12% of those operatively treated. In this study, 17% of patients reported severe or extreme pain on the EQ5D‐5L pain score with movement at 1 week. Severe pain persisted in 13% at 4 weeks and 16% at 3 months, though it was derived from a small surviving cohort.

In the FRAIL‐HIP non‐operative cohort, 88% of patients scored ≥ 4 on the PACSLAC‐D at 1 week and 64% at 4 weeks [4] compared with 40% (15/36) and 44% (8/18) in our cohort; this suggests better pain relief, which was more comparable with the operative cohort (67% at 1 week and 28% at 4 weeks). Opioid use was common in the FRAIL‐HIP study. Patients in the non‐operative cohort required a median of 68 mg daily OME at week 1, 25 mg at 4 weeks, and 28 mg at 3 months [4]. In our cohort, opioid use was less after 1 week (median OME 30 mg), similar at 30 days (median OME 30 mg) and lower (median OME 15 mg) after 3 months. Persistent opioid use in both cohorts may reflect residual pain or under‐recognition of tapering needs. Opioid use was lower in the operative group, with a median OME of 17 mg after 1 week and 4 mg at 4 weeks and 3 months [4].

Functional outcomes were more favourable in our cohort. After 1 week, 47% achieved bed–chair transfer or steps, rising to 63% (19/30) at 30 days and (17/19) 90% at 3 months. In contrast, 89% of the non‐operative cohort in the FRAIL‐HIP remained bedbound [4]. Nevertheless, the EQ5D‐5L showed continued limitations in walking and self‐care in both cohorts. Further analysis with matched cohorts may help determine whether, and to what extent, differences in the experienced quality of life exist.

Although chemical denervation of the hip capsule is a relatively new technique, it is regarded as an effective, minimally invasive and theoretically safe option for pain relief. Due to the absence of an evidence based, minimal invasive alternative in this vulnerable population and pain being a major issue, randomisation would mean withholding possible pain relief. After consultation with the medical ethics committee, it was considered ethically unjustifiable, and a large multicentred prospective database study was proposed instead.

Only the phenol concentration (6%) was standardised across sites; therefore, procedural variation may have occurred. Although all interventions were ultrasound‐guided, images were not stored or reviewed, meaning procedures performed incorrectly cannot be ruled out. The selective targeting of the articular branches towards the anterior hip capsule with multiple needle placements was proposed in previous studies on chemical denervation, to limit spread to the afferent nerves [18, 27]. Recent studies support chemical neurolysis via the PENG block in patients with acute and chronic hip pain, justifying the use of this technique in patients with a hip fracture [20, 39].

Complications may also be underestimated, as no additional diagnostics were performed. Loss to follow up due to refusal was low, however, data beyond 30 days and 3 months were limited by high mortality.

Despite these limitations, this cohort study supports realistic informed consent and improved communication with patients and families, which is crucial in this vulnerable population [21]. The decision for non‐operative management should be balanced carefully against operative management, as stabilisation offers effective analgesia with mobility within the palliative care context, especially when mortality is not expected in the short term [40]. Anterior capsule neurolysis – alongside other systemic pain relief measures – could serve as a minimally invasive alternative for patients who are immobile or those unlikely to regain mobility.

Addition of posterior hip pericapsular neurolysis seems effective in patients with residual pain, due to nociceptive innervation of the posterior hip capsule in a subset of patients [27]. The median discharge time of 3 days offers a practical window to add posterior hip pericapsular neurolysis when a PENG block is insufficient, but the required repositioning may cause discomfort and need for sedation. Future research should evaluate the benefit of posterior hip pericapsular neurolysis as an adjunct in non‐operative hip fracture care.

Phenol is neurolytic at concentrations above 3.3–5%, with immediate local anaesthetic effect and neurolysis developing over 3–7 days. Dilution below 5% might alter this effect [41]. Our median phenol 6% volume was 10 ml. Higher volumes correlated with higher treatment satisfaction at 24 h, but not at 1 week. In > 10% of the procedures, > 5 ml of other additives was used, potentially reducing the neurolytic effect of phenol. Lidocaine use was associated with lower satisfaction, though numbers were small, possibly reflecting operator preference and could be an incidental finding, hence, lesser treatment satisfaction in one centre. Standardising the procedure, with explicit guidance on dilution when large volumes of other fluids are administered and structured operator training, could reduce practice variability and improve outcomes.

The PENG block is a minimally invasive technique that can be performed at the bedside without mobilising or transporting the patient, thus increasing comfort without the need for sedatives. It enables the use in outpatient clinics or nursing homes, leading to reduction in patient burden by preventing a potential painful and expensive transport to hospital.

This study has established that the PENG block with phenol 6% is an effective minimally invasive procedure for pain management in addition to opioid‐based pain relief in patients living with frailty, when surgery is not chosen. Adverse events appear to be uncommon despite anticoagulant use in a substantial number of patients. A graphical overview is given in online Supporting Information Figure S2.

## Supporting information


**Appendix S1.** Dutch‐PENG registry collaborators from the participating hospitals.

**Figure S1.** Cumulative survival in days.
**Figure S2.** The PENG block with phenol in non‐operative management for patients with a hip fracture.

**Table S1.** Mortality rate.
**Table S2.** Day of death per patient.
**Table S3.** Univariate regression analysis.

## References

[1] Lommerse MI , Willems HC , van Dieren S , Bloemers FW , Schuijt HJ , van Embden D . Increasing incidences of acetabular, pelvic, and proximal femur fractures in the Netherlands. Injury 2025; 56: 112322. 10.1016/j.injury.2025.112322. 40198969

[2] Sing CW , Lin TC , Bartholomew S , et al. Global epidemiology of hip fractures: secular trends in incidence rate, post‐fracture treatment, and all‐cause mortality. J Bone Miner Res 2023; 38: 1064–1075. 10.1002/jbmr.4821. 37118993

[3] Smith T , Pelpola K , Ball M , Ong A , Myint PK . Pre‐operative indicators for mortality following hip fracture surgery: a systematic review and meta‐analysis. Age Ageing 2014; 43: 464–471. 10.1093/ageing/afu065. 24895018

[4] Loggers SAI , Willems HC , Van Balen R , et al. Evaluation of quality of life after nonoperative or operative management of proximal femoral fractures in frail institutionalized patients: the FRAIL‐HIP study. JAMA Surg 2022; 157: 424–434. 10.1001/jamasurg.2022.0089. PMC8892372 35234817

[5] Loggers SAI , Van Lieshout EMM , Joosse P , Verhofstad MHJ , Willems HC . Prognosis of nonoperative treatment in elderly patients with a hip fracture: a systematic review and meta‐analysis. Injury 2020; 51: 2407–2413. 10.1016/j.injury.2020.08.027. 32907702

[6] Wurdemann FS , Krijnen P , van Zwet EW , et al. Trends in data quality and quality indicators 5 years after implementation of the dutch hip fracture audit. Eur J Trauma Emerg Surg 2022; 48: 4783–4796. 10.1007/s00068-022-02012-y. PMC9712331 35697872

[7] Bremen HV , Kroes T , Seppala LJ , Gans EA , Hegeman JH , van der Velde N , Willems HC . Variability in care pathways for hip fracture patients in The Netherlands. J Clin Med 2024; 13: 13. 10.3390/jcm13164589. PMC11355027 39200731

[8] Zeelenberg ML , Oosterwijk PC , Willems HC , et al. Shared decision‐making for non‐operative management versus operative management of hip fractures in selected frail older adults with a limited life expectancy: a protocol for a nationwide implementation study. BMJ Open 2024; 14: e083429. 10.1136/bmjopen-2023-083429. PMC11029367 38631829

[9] van der Steen JT , Engels Y , Touwen DP , Kars MC , Reyners AKL , van der Linden YM , Korfage IJ . Advance care planning in The Netherlands. Z Evid Fortbild Qual Gesundhwes 2023; 180: 133–138. 10.1016/j.zefq.2023.06.003. 37482528

[10] Xu J , Mehr DR , Perry M , et al. Advance care planning with people living with dementia: ethical considerations of physicians in the United States and The Netherlands. J Gerontol B Psychol Sci Soc Sci 2025; 80: gbaf155. 10.1093/geronb/gbaf155. PMC12507472 40839070

[11] Van Bremen HE , Lommerse MI , Hegeman JH , et al. Use of neurolytic nerve blocks and mortality in nonoperative management of hip fracture patients: a nationwide population study in The Netherlands. Bone Joint J 2026; 108‐B: 8. 10.1302/0301-620X.108B8.BJJ-2025-1465.R1. 42537998

[12] Benyamin R , Trescot AM , Datta S , et al. Opioid complications and side effects. Pain Physician 2008; 11: S105–S120. 10.36076/ppj.2008/11/s105. 18443635

[13] Guay J , Kopp S . Peripheral nerve blocks for hip fractures in adults. Cochrane Database Syst Rev 2020; 11: CD001159. 10.1002/14651858.CD001159.pub3. PMC8130997 33238043

[14] Handoll HH , Parker MJ . Conservative versus operative treatment for hip fractures in adults. Cochrane Database Syst Rev 2008; 2010: CD000337. 10.1002/14651858.CD000337.pub2. PMC12582187 18646065

[15] Giron‐Arango L , Peng PWH , Chin KJ , Brull R , Perlas A . Pericapsular nerve group (PENG) block for hip fracture. Reg Anesth Pain Med 2018; 43: 859–863. 10.1097/AAP.0000000000000847. 30063657

[16] Short AJ , Barnett JJG , Gofeld M , Baig E , Lam K , Agur AMR , Peng PWH . Anatomic study of innervation of the anterior hip capsule: implication for image‐guided intervention. Reg Anesth Pain Med 2018; 43: 186–192. 10.1097/AAP.0000000000000701. 29140962

[17] Giron‐Arango L , Peng P . Pericapsular nerve group (PENG) block: what have we learned in the last 5 years? Reg Anesth Pain Med 2025; 50: 402–409. 10.1136/rapm-2024-105427. 38724271

[18] Kwun‐Tung Ng T , Chan WS , Peng PWH , Sham P , Sasaki S , Tsui HF . Chemical hip denervation for inoperable hip fracture. Anesth Analg 2020; 130: 498–504. 10.1213/ANE.0000000000004172. 30985383

[19] Rocha Romero A , Carvajal Valdy G , Lemus AJ . Ultrasound‐guided pericapsular nerve group (PENG) hip joint phenol neurolysis for palliative pain. Can J Anaesth 2019; 66: 1270–1271. 10.1007/s12630-019-01448-y. 31301017

[20] Kroes T , Wijnen HH , Bos K , et al. Chemical hip denervation using phenol via pericapsular nerve group (PENG) block in palliative non‐operative management for frail older hip fracture patients: a multicenter retrospective cohort study. J Clin Anesth 2025; 105: 111872. 10.1016/j.jclinane.2025.111872. 40435923

[21] Kroes T , van Breugel JM , Smits R , Jan Schuijt H , van der Velde D , Willems HC , Dutch PENG Study Group . Experience with hip denervation in non‐operative hip fracture care for frail older patients in The Netherlands: an interview study. BMJ Open 2025; 15: e095738. 10.1136/bmjopen-2024-095738. PMC12067832 40345698

[22] Reijneveld SA , Spijker J , Dijkshoorn H . Katz' ADL index assessed functional performance of Turkish, Moroccan, and Dutch elderly. J Clin Epidemiol 2007; 60: 382–388. 10.1016/j.jclinepi.2006.02.022. 17346613

[23] Herdman M , Gudex C , Lloyd A , et al. Development and preliminary testing of the new five‐level version of EQ‐5D (EQ‐5D‐5L). Qual Life Res 2011; 20: 1727–1736. 10.1007/s11136-011-9903-x. PMC3220807 21479777

[24] Charlson ME , Pompei P , Ales KL , MacKenzie CR . A new method of classifying prognostic comorbidity in longitudinal studies: development and validation. J Chronic Dis 1987; 40: 373–383. 10.1016/0021-9681(87)90171-8. 3558716

[25] Fuchs‐Lacelle S , Hadjistavropoulos T . Development and preliminary validation of the pain assessment checklist for seniors with limited ability to communicate (PACSLAC). Pain Manag Nurs 2004; 5: 37–49. 10.1016/j.pmn.2003.10.001. 14999652

[26] Patrick DL , Engelberg RA , Curtis JR . Evaluating the quality of dying and death. J Pain Symptom Manag 2001; 22: 717–726. 10.1016/s0885-3924(01)00333-5. 11532585

[27] Ng TK , Peng P , Chan WS . Posterior hip pericapsular neurolysis (PHPN) for inoperable hip fracture: an adjunct to anterior hip pericapsular neurolysis. Reg Anesth Pain Med 2021; 46: 1080–1084. 10.1136/rapm-2021-103023. 34686579

[28] Kim SJ , Park HS , Lee DW . Outcome of nonoperative treatment for hip fractures in elderly patients: a systematic review of recent literature. J Orthop Surg (Hong Kong) 2020; 28: 2309499020936848. 10.1177/2309499020936848. 32638635

[29] van Bremen HE , Seppala LJ , Hegeman JH , van der Velde N , Willems HC , Dutch Hip Fracture Audit G . Characteristics and outcomes of nonoperatively managed patients with hip fracture using the Dutch Hip Fracture Audit. J Orthop Trauma 2024; 38: 265–272. 10.1097/BOT.0000000000002778. PMC11017835 38259058

[30] Kietaibl S , Ferrandis R , Godier A , et al. Regional anaesthesia in patients on antithrombotic drugs: joint ESAIC/ESRA guidelines. Eur J Anaesthesiol 2022; 39: 100–132. 10.1097/EJA.0000000000001600. 34980845

[31] Pascarella G , Costa F , Gargano F , Cataldo R , Agro FE , Carassiti M . Pericapsular nerve group block in patients on antithrombotic drugs: a reply to ESAIC/ESRA guidelines. Eur J Anaesthesiol 2023; 40: 454–455. 10.1097/EJA.0000000000001800. 36924169

[32] Gargano F , Migliorelli S , Pascarella G , et al. A randomized clinical trial comparing different combination of peripheral nerve blocks for intraoperative analgesia in patients on antithrombotic drugs undergoing hip fracture surgery: pericapsular nerve group (PENG) block versus femoral and obturator nerve block. Minerva Anestesiol 2024; 91: 524–532. 10.23736/S0375-9393.24.18534-3. 39656148

[33] Dutch Society of Anaesthesiology . [Neuraxis block and anticoagulation]. 2026. https://richtlijnendatabase.nl/richtlijn/neuraxisblokkade_en_antistolling/startpagina_‐_neuraxisblokkade_en_antistolling.html. (accessed 20/05/2026).

[34] Leurcharusmee P , Kantakam P , Intasuwan P , et al. Cadaveric study investigating the femoral nerve‐sparing volume for pericapsular nerve group (PENG) block. Reg Anesth Pain Med 2023; 48: 549–552. 10.1136/rapm-2023-104419. 37028817

[35] Harman H , Walton TJ , Chan G , Stott P , Ricketts DM , Rogers BA . Predicting 30‐day mortality after hip fracture: the G4A calibrated prognostic tool. Hip Int 2022; 32: 820–825. 10.1177/1120700021998959. 33755498

[36] Winfield J , McNicoll L , Moppett IK . Epidemiology, patient outcome and complications after non‐operative management of hip fracture: a systematic review. Anaesthesia 2025; 80: 1397–1413. 10.1111/anae.16732. PMC12519934 40855691

[37] Davies C , Harvey L , Mitchell R , Sarkies M , Navarre P , Wall C . What is the place for non‐operative management of neck of femur fractures – analysis of the Australia and New Zealand Hip Fracture Registry (ANZHFR). Hip Int 2026; 36: 11207000251408435. 10.1177/11207000251408435. 41700326

[38] Wijnen HH , Schmitz PP , Es‐Safraouy H , Roovers LA , Taekema DG , Van Susante JLC . Nonoperative management of hip fractures in very frail elderly patients may lead to a predictable short survival as part of advance care planning. Acta Orthop 2021; 92: 728–732. 10.1080/17453674.2021.1959155. PMC8635672 34319206

[39] Reysner M , Reysner T , Kowalski G , Mularski A , Daroszewski P , Wieczorowska‐Tobis K . Chemical ablation of pericapsular nerve group with 95% ethanol for pain relief and quality of life in patients with hip osteoarthritis: a prospective, double‐blinded, randomised, controlled trial. Br J Anaesth 2025; 135: 382–389. 10.1016/j.bja.2025.04.045. 40480912

[40] Foss NB , Overgaard S . Too unfit not to have surgery: is non‐operative care for hip fracture patients a viable option? Anaesthesia 2025; 80: 1308–1311. 10.1111/anae.16735. 40856076

[41] Creemers JHA , Smits RJH , Patel AM , van Dongen RTM , Kramers CK , Bos JM , van der Wal SEI . Phenol neurolysis in pain and palliative medicine. Pain Pract 2026; 26: e70153. 10.1111/papr.70153. PMC13077028 41978248

