# Supplementary material for: Pericapsular nerve group block with phenol for non‐operative hip fracture management in patients living with frailty: a prospective multicentre cohort study
Source: Anaesthesia. 2026 Sep 8;81(12):1673–85. doi: 10.1111/anae.70346 (PMC13743663; doi:10.1111/anae.70346)
Supplement: Supplementary file 1 [file ANAE-81-1673-s001.pdf]

Appendix S1 The list of the Dutch-PENG registry collaborators from the participating hospitals

Last name	First name and initials	Academic degrees	Institution	Department	City	Role
Roukema	Gert R.	MD	Maasstad hospital	Surgery	Rotterdam	Collaborator
Van der Stap	Dawi	MD	Maasstad hospital	Geriatrics	Rotterdam	Collaborator
Koopman	J.S.H.A. (Seppe)	MD	Maasstad hospital	Anaesthesiology	Rotterdam	Collaborator
Spaetgens	Bart P.A.	MD PhD	Maastricht UMC	Internal medicine, geriatrics	Maastricht	Collaborator
Brüggemann	Renée	MD PhD	Maastricht UMC	Internal medicine, geriatrics	Maastricht	Collaborator
Lefel	Nicole	MD	Maastricht UMC	Anaesthesiology, pain- and palliative medicine	Maastricht	Collaborator
Vugts	Manon	MD	Radboudumc	Anaesthesiology, pain- and palliative medicine	Nijmegen	Collaborator
Noorda	Nikki M.F.	MD PhD	Dijklander Hospital	Geriatrics	Hoorn	Collaborator
Van Dongen	Vincent	MD	Zuyderland hospital	Anaesthesiology and pain medicine	Heerlen	Collaborator
Krot	Hidde	MD	Zuyderland hospital	Anaesthesiology and pain medicine	Heerlen	Collaborator
Grupa	Nicole	MD	Zuyderland hospital	Anaesthesiology and pain medicine	Heerlen	Collaborator
Hoofwijk	Daisy	MD PhD	Zuyderland hospital	Anaesthesiology and pain medicine	Heerlen	Collaborator
Timmerman	Léon	MD PhD	St. Antonius hospital	Anaesthesiology	Nieuwegein	Collaborator
