# Supplementary material for: Pericapsular nerve group block with phenol for non‐operative hip fracture management in patients living with frailty: a prospective multicentre cohort study
Source: Anaesthesia. 2026 Sep 8;81(12):1673–85. doi: 10.1111/anae.70346 (PMC13743663; doi:10.1111/anae.70346)
Supplement: Supplementary file 2 [file ANAE-81-1673-s002.pdf]

Figure S1 Cumulative survival (vertical) in days (horizontal).
Light blue, women; dark blue, men.

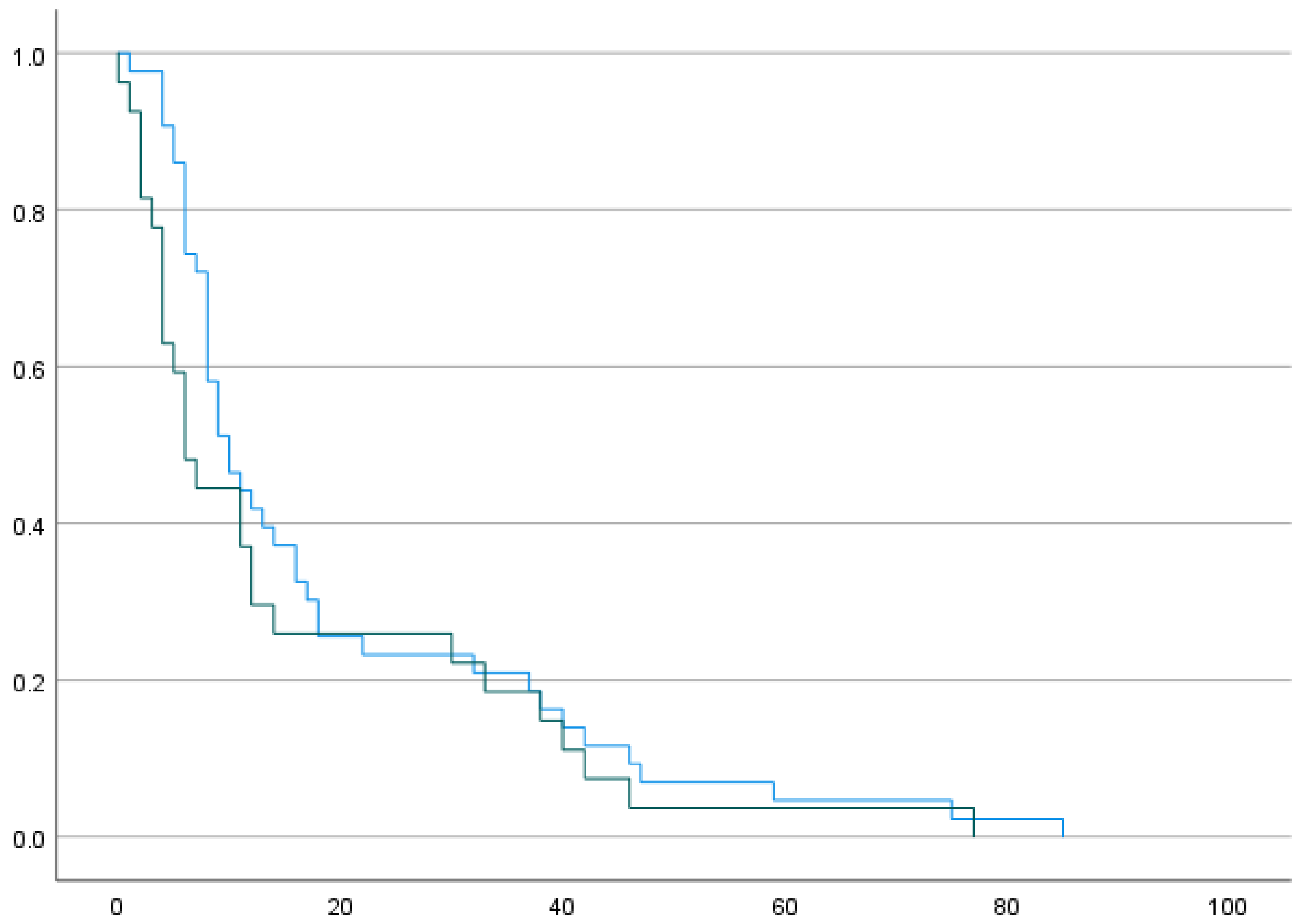

Figure S2

The PENG block with phenol in non-operative management for patients with a hip fracture

Dutch PENG Study

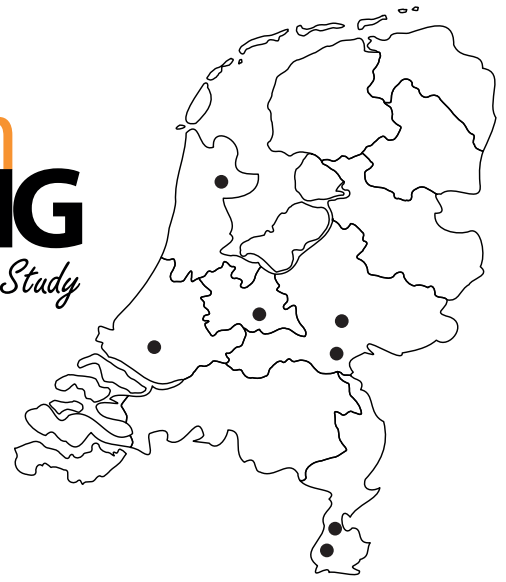

103 patients

86.6 mean age

61% women

78% cognitive impairment

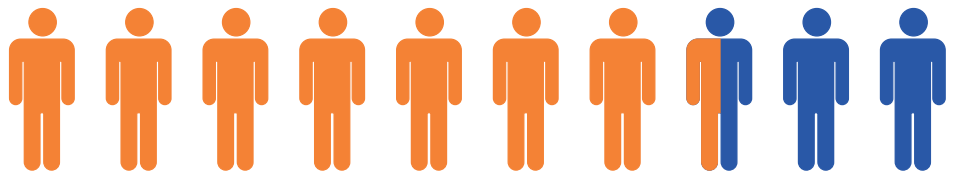

74% femoral neck fracture
26% trochanteric fracture

Intervention

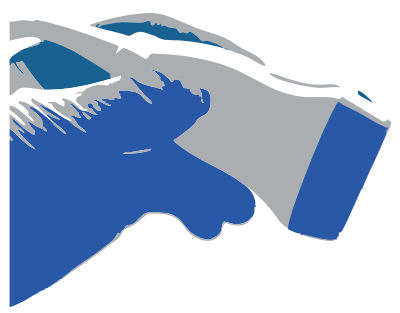

10.4 mean quantity in ml of phenol 6% was used

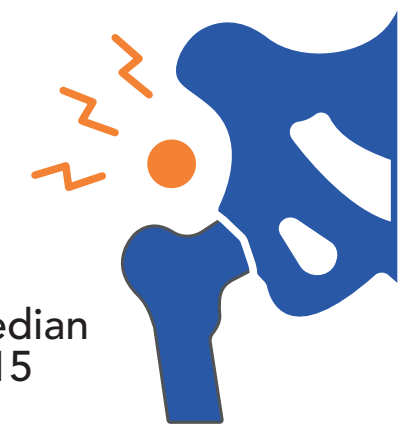

2 mean quantity in ml was used for hydrodissectom

2 patients experienced discomfort due to the intervention

2 was the median NRS after 15 minutes

Mortality

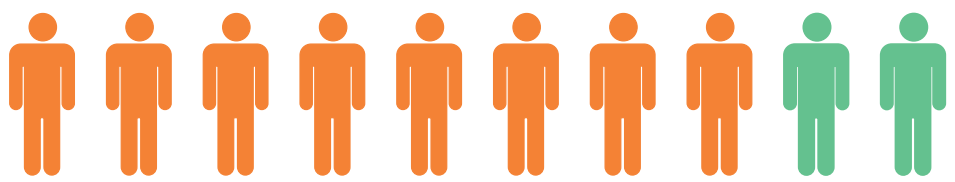

79% mortality at 3 months

61% 30-day mortality

9 median survival days

Results on pain

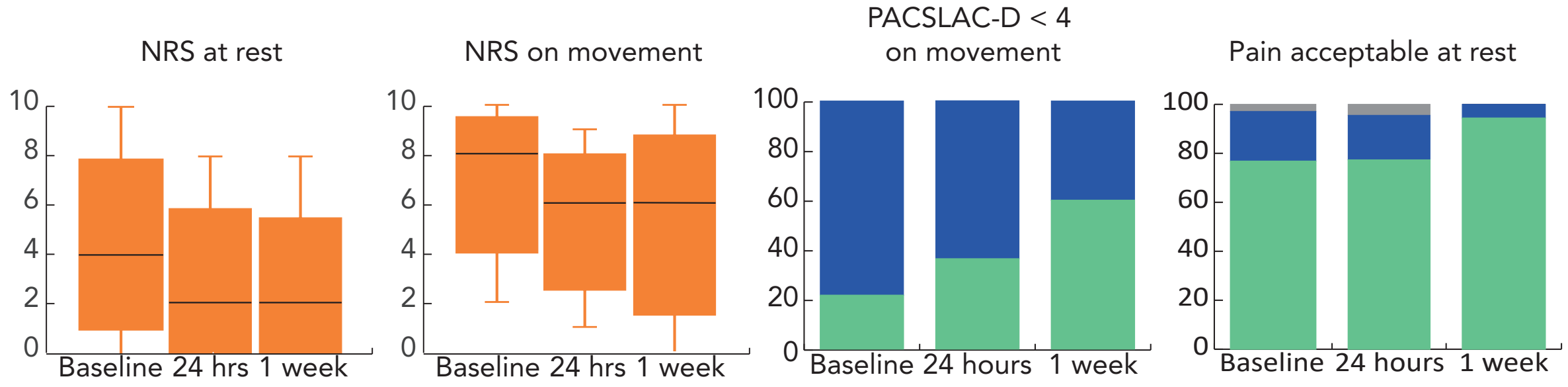

1 week	30 days	3 months

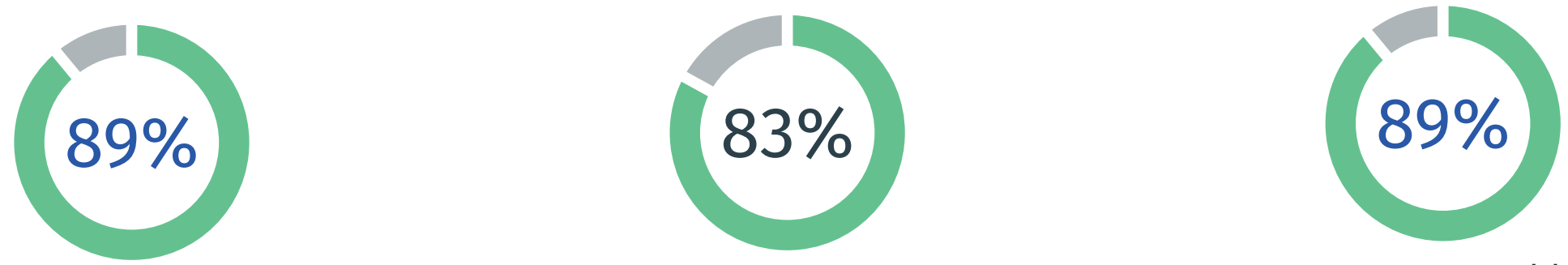

No or acceptable pain during washing and dressing (1 week)

No or acceptable pain during washing and dressing (30 days)

No or acceptable pain during washing and dressing (3 months)

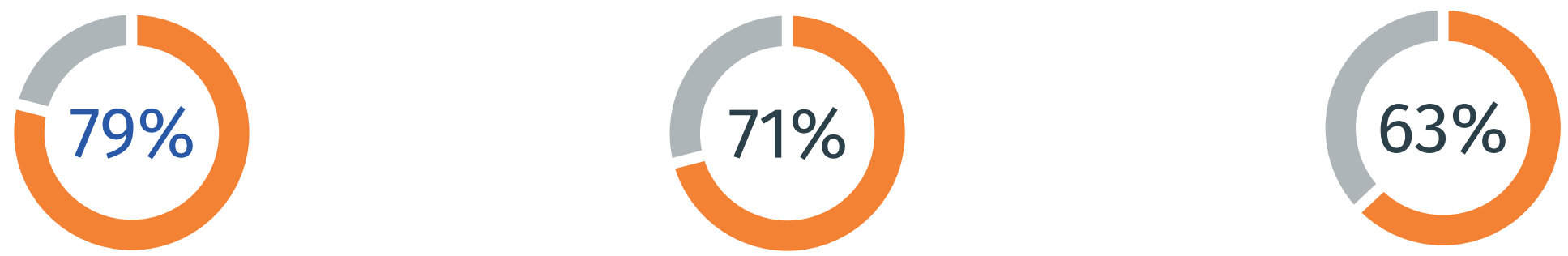

opioid use | opioid use | opioid use

30 OME | **30** OME | **15** OME

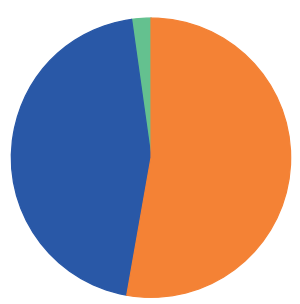

Bedbound 53%
Bed-chair 45%
Few steps 2%

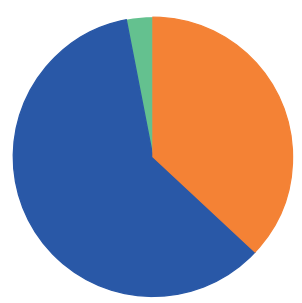

Bedbound 37%
Bed-chair 60%
Few steps 3%

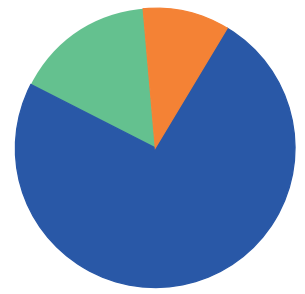

Bedbound 10%
Bed-chair 74%
Few steps 16%
