# Supplementary material for: Pericapsular nerve group block with phenol for non‐operative hip fracture management in patients living with frailty: a prospective multicentre cohort study
Source: Anaesthesia. 2026 Sep 8;81(12):1673–85. doi: 10.1111/anae.70346 (PMC13743663; doi:10.1111/anae.70346)
Supplement: Supplementary file 3 [file ANAE-81-1673-s003.docx]

|  | **N = 91** |
| --- | --- |
| < 1 week | 28 (31%) |
| < 30 days | 56 (62%) |
| < 3 months | 72 (79%) |
| Survival; days | 9 (5.8 – 24 [0-85]) |

**Table S1** Mortality rate. Values are number (proportion) or median (IQR [range]).

**Table S2** Day of death per patient. Values are numbers.

| Day | N |
| --- | --- |
| 0 | 1 |
| 1 | 2 |
| 2 | 3 |
| 3 | 1 |
| 4 | 7 |
| 5 | 3 |
| 6 | 8 |
| 7 | 2 |
| 8 | 6 |
| 9 | 3 |
| 10 | 2 |
| 11 | 3 |
| 12 | 3 |
| 13 | 1 |
| 14 | 2 |
| 16 | 2 |
| 17 | 1 |
| 18 | 2 |
| 22 | 1 |
| 30 | 1 |
| 32 | 1 |
| 33 | 1 |
| 37 | 1 |
| 38 | 2 |
| 40 | 2 |
| 42 | 2 |
| 46 | 2 |
| 47 | 1 |
| 59 | 1 |
| 75 | 1 |
| 77 | 1 |
| 85 | 1 |
| Missing | 2 |
| Total | 72 |

**Table S3** Univariate regression analysis. Values are number (proportion), effect size (95% confidence interval) or p-value.
* refers to the intercept.

|  | **EQ5D-5L**  **1 week** |  |  | **Treatment satisfaction 24 hours** |  |  | **Treatment satisfaction 1 week** |  |  |
| --- | --- | --- | --- | --- | --- | --- | --- | --- | --- |
|  | Value  N (%) | β (95% CI) | p-value | Value  N (%) | β (95% CI) | p-value | Value  N (%) | β (95% CI) | p-value |
| **Centre** |  |  |  |  |  |  |  |  |  |
| 1 * | 6 (9%) | 1.87  (0.55 – 3.20) | Ref | 5 (6%) | 6.40 (4.42 – 8.38) | Ref | 6 (8%) | 5.83 (3.95 – 7.72) | Ref |
| 2 | 16 (23%) | -0.20  (-1.19 – 0.78) | 0.68 | 29 (34%) | -1.43 (-3.58 – 0.71) | 0.19 | 16 (22%) | 0.17 (-2.05 – 2.38) | 0.88 |
| 3 | 12 (17%) | -0.12  (-1.13 – 0.88) | 0.80 | 15 (17%) | 0.73 (-1.55 – 3.02) | 0.53 | 12 (17%) | 1.50 (-0.81 – 3.81) | 0.20 |
| 4 | 10 (14%) | 0.35 (-0.72 – 1.42) | 0.52 | 9 (11%) | -0.73 (-3.20 – 1.74) | 0.56 | 11 (15%) | 0.80 (-1.54 – 3.15) | 0.50 |
| 5 | 9 (13%) | -0.32  (-1.38 – 0.74) | 0.55 | 7 (8%) | -3.26 (-5.85 – -0.66) | **0.014** | 9 (12,5%) | -0.72 (-3.16 – 1.71) | 0.56 |
| 6 | 9 (13%) | -0.290  (-1.39 – 0.81) | 0.60 | 13 (15%) | 0.29 (-2.04 – 2.62) | 0.80 | 9 (12,5%) | -0.056 (-2.49 – 2.38) | 0.96 |
| 7 | 8 (11%) | 0.41  (-0.72 – 1.54) | 0.47 | 8 (9%) | 1.35 (-1.18 –3.88) | 0.29 | 9 (12,5%) | -0.056 (-2.49 – 2.38) | 0.96 |
| **Anticoagulant** |  |  |  |  |  |  |  |  |  |
| Direct oral anticoagulants * | 10 (14%) | 2.10 (1.01 – 3.18) | Ref | 14 (16%) | 4.993 (3.63 – 6.23) | Ref | 11 (15%) | 5.46 (4.06 – 6.85) | Ref |
| Vitamin K antagonist | 6 (9%) | -0.29 (-1.33 – 0.74) | 0.58 | 10 (12%) | 0.47 (-1.54 – 2.48) | 0.64 | 6 (9%) | 0.71 (-1.64 – 3.07) | 0.55 |
| Platelet aggregation inhibitors | 20 (29%) | -0.23 (-1.02 – 0.56) | 0.56 | 20 (24%) | 1.92 (0.23 – 3.61 | **0.026** | 20 (28%) | 0.65 (-1.10 – 2.39) | 0.46 |
| Low Molecular Weight Heparin | 2 (3%) | 0.32 (-1.23 – 1.88) | 0.68 | 4 (5%) | 0.07 (-2.68 – 2.82) | 0.96 | 2 (3%) | -0.46 (-4.02 – 3.11) | 0.80 |
| None | 31 (45%) | -0.22 (-0.94 – 0.49) | 0.54 | 37 (44%) | 0.88 (-0.64 – 2.41) | 0.25 | 32 (45%) | 0.89 (-0.73 – 2.51) | 0.28 |
| **Gender** |  |  |  |  |  |  |  |  |  |
| Female* | 47 (67%) | 1.86 (1.09 – 2.64) | Ref | 50 (58%) | 6.16 (5.46 – 6.86) | Ref | 49 (68%) | 6.14 (5.48 – 6.81) | Ref |
| Male | 23 (33%) | 0.16 (-0.34 – 0.66) |  | 36 (42%) | -0.69 (-1.77 – 0.39) | 0.21 | 23 (32%) | -0.012 (-1.12 – 1.63) | 0.98 |
| **CCI** | 70 | 1.70 (0.83 –2.56) | Ref | 86 | 6.10 (5.01 – 7.10) | Ref | 72 | 6.19 (5.14 – 7.24) | Ref |
|  |  | 0.05 (-0.04 – 0.14) | 0.30 |  | -0.69 (-1.77 – 0.39) | 0.21 |  | -0.012 (-0.22 – 0.20 | 0.91 |
| **preOME** | 11 | 2.23 (0.22 – 4.23) | Ref | 13 | 7.38 (4.96 – 9.81) | Ref | 11 | 5.37 (2.12 – 8.62) | Ref |
|  |  | 0.004 (-0.02 – 0.02) | 0.69 |  | -0.03 (-0.06 – -0.002) | **0.04** |  | 0.009 (-0.04 – 0.06) | 0.71 |
| **Dementia** |  |  |  |  |  |  |  |  |  |
| No * | 27 (39%) | 2.15 (1.31 –2.99) | Ref | 34 (40%) | 5.26 (4.43 – 6.10) | Ref | 29 (40%) | 5.90 (5.04 – 6.76) | Ref |
| Yes | 43 (61%) | -0.31(-0.79 – 0.17) | 0.20 | 52 (60%) | 1.00 (-0.07 – 2.08) | 0.068 | 43 (60%) | 0.41 (-0.71 – 1.52) | 0.47 |
| **Cognitive disorders** |  |  |  |  |  |  |  |  |  |
| No* | 16 (24%) | 2.25 (1.26-3.24) | Ref | 19 (23%) | 5.32 (4.18 – 6.45) | Ref | 17 (25%) | 6.47 (5.65 – 7.29) | Ref |
| Yes | 51 (76%) | 0.01 (0.001-0.014) | **0.02** | 65 (77%) | 0.64 (-0.65 – 1.93) | 0.327 | 52 (75%) | -0.01 (-0.02 – 0.005) | 0.176 |
| **Immobile or FAC < 2** |  |  |  |  |  |  |  |  |  |
| No* | 49 (70) | 1.90 (1.11 – 2.69) | Ref | 64 (74%) | 5.72 (5.10 – 6.34) | Ref | 51 (71%) | 6.28 (5.63 – 6.92) | Ref |
| Yes | 21 (30) | 0.04 (-0.48 – 0.56) | 0.88 | 22 (26%) | 0.60 (-0.63 – 1.83) | 0.33 | 21 (29%) | -0.47 (-1.67 – 0.74) | 0.44 |
| **Fracture type** |  |  |  |  |  |  |  |  |  |
| Trochanteric* | 22 (31%) | 2.28 (1.42 – 3.14) | Ref | 23 (27%) | 4.96 (3.94 – 5.97) | Ref | 22 (31%) | 5.77 (4.79 – 6.76) | Ref |
| Femoral neck | 48 (69%) | -0.42 (-0.92 – 0.08) | 0.10 | 63 (73%) | 1.25 (0.06 – 2.44) | **0.039** | 50 (69%) | 0.53 (-0.66 – 1.71) | 0.377 |
| **Difficulty level intervention** |  |  |  |  |  |  |  |  |  |
| Easy* | 44 (63%) | 1.94 (1.15 – 2.72) | Ref | 59 (69%) | 5.48 (4.84 – 6.11) | Ref | 46 (64%) | 5.98 (5.30 – 6.65) | Ref |
| Moderate | 24 (34%) | -0.10 (-0.59 – 0.40) | 0.70 | 25 (29%) | 1.25 (0.08 –2.41) | **0.036** | 24 (33%) | 0.65 (-0.51 – 1.80) | 0.27 |
| Difficult | 2 (3%) | 0.79 (-0.63 – 2.21) | 0.27 | 2 (2%) | 1.52 (-1.98 –5.03) | 0.39 | 2 (3%) | -1.98 (-5.29 – 1.33) | 0.24 |
| **Phenol 6% mL** | 70 | 1.51 (0.17 – 2.85) | Ref | 85 | 3.91 (1.70 – 6.12) | Ref | 72 | 6.36 (4.00 – 8.72) | Ref |
|  |  | 0.04 (-0.06 – 0.13) | 0.44 |  | 0.19 (-0.021 – 0.40) | 0.08 |  | -0.02 (-0.24 – 0.20) | 0.86 |
| **Additive** |  |  |  |  |  |  |  |  |  |
| None*  0,9% Saline  Lidocaine  Ropivacaine  Bupivacaine | 16 (23%)  21 (30%)  5 (7%)  26 (37%)  2 (3%) | 1.81 (1.02 – 2.61  0.18 (-0.43 – 0.79)  1.40 (0.47 – 2.34)  -0.16 (-0.74 – 0.43)  -0.62 (-2.00 – 0.75) | Ref  0.56  **0.04**  0.59  0.37 | 17 (20%)  23 (27%)  7 (8%)  36 (42%)  3 (3%) | 6.41 ( 5.33 – 7.49)  0.72 (-0.71 – 2.14)  -1.98 (-3.98 – 0.02)  -1.58 (-2.89 – -0.27)  2.59 (-0.20 – 5.38) | Ref  0.32  0.052  **0.019**  0.07 | 16 (22%)  23 (32%)  5 (7%)  26 (36%)  2(3%) | 6.13 (5.06 – 7.18)  0.92 ( -0.46 – 2.30  -3.13 (-5.30 – -0.95)  -0.32 (-1.67 – 1.03)  1.88 (-1.31 – 5.06) | Ref  0.19  **0.005**  0.64  0.24 |
| **Liquid in mL** | 70 | 1.88 (1.11 – 2.65) | Ref | 85 | 6.55 (5.88 – 7.22) | Ref | 72 | 6.59 (5.93 – 7.25) | Ref |
|  |  | 0.027 (-0.05 – 0.1) | 0.47 |  | -0.25 (-0.42 – -0.09) | **0.003** |  | -0.19 (-0.35 – -0.02) | 0.027 |

**CCI, Charlson Comorbidity Index; preOME, Oral Morphine Equivalent in mg per 24h before hip fracture**
